# Automated tear film break-up time measurement for dry eye diagnosis using deep learning

**DOI:** 10.1038/s41598-024-62636-5

**Published:** 2024-05-22

**Authors:** Fatima-Zahra El Barche, Anas-Alexis Benyoussef, Mostafa El Habib Daho, Antonin Lamard, Gwenolé Quellec, Béatrice Cochener, Mathieu Lamard

**Affiliations:** 1 LaTIM UMR 1101, Inserm, Brest, France; 2 Université de Bretagne Occidentale, Brest, France; 3 Ophtalmology Departement, CHRU Brest, Brest, France

**Keywords:** Artificial intelligence, Deep learning, Dry eye disease, Dual task learning, Siamese network, Tear film breakup time, Diseases, Medical research

## Abstract

In the realm of ophthalmology, precise measurement of tear film break-up time (TBUT) plays a crucial role in diagnosing dry eye disease (DED). This study aims to introduce an automated approach utilizing artificial intelligence (AI) to mitigate subjectivity and enhance the reliability of TBUT measurement. We employed a dataset of 47 slit lamp videos for development, while a test dataset of 20 slit lamp videos was used for evaluating the proposed approach. The multistep approach for TBUT estimation involves the utilization of a Dual-Task Siamese Network for classifying video frames into tear film breakup or non-breakup categories. Subsequently, a postprocessing step incorporates a Gaussian filter to smooth the instant breakup/non-breakup predictions effectively. Applying a threshold to the smoothed predictions identifies the initiation of tear film breakup. Our proposed method demonstrates on the evaluation dataset a precise breakup/non-breakup classification of video frames, achieving an Area Under the Curve of 0.870. At the video level, we observed a strong Pearson correlation coefficient (r) of 0.81 between TBUT assessments conducted using our approach and the ground truth. These findings underscore the potential of AI-based approaches in quantifying TBUT, presenting a promising avenue for advancing diagnostic methodologies in ophthalmology.

## Introduction

Dry eye disease (DED) is a prevalent and chronic ocular condition that significantly impacts the quality of life, yet it is frequently underestimated and underdiagnosed in clinical practice^1^. Recognized as a major public health issue, the prevalence of DED varies widely from 5 to 50%, influenced by environmental factors and lifestyle changes^1^. This multifactorial disorder is characterized by a range of distressing symptoms, including ocular discomfort, visual disturbances, tear film instability, elevated tear osmolarity, and inflammation of the ocular surface, leading to persistent discomfort and reduced visual acuity, affecting daily activities of those afflicted^1^. According to Craig et al.^2^, dry eye can be classified into two primary categories: aqueous deficient and evaporative. The epidemiology of DED is challenging due to the lack of standardized worldwide definitions. Further research is needed to advance our understanding of dry eye, improve diagnostic methods, develop effective therapies, and individualize diagnostic approaches. The complexities in diagnosing DED stem from its diverse presentation and the subjective nature of current testing methods. At present, comprehensive approaches include questionnaires, risk factor analysis, patient history, anterior eye examination, and assessing the ocular surface through clinical tests like the tear film break-up time (TBUT) test. This test is crucial for assessing tear film stability and identifying evaporative dry eyes. A TBUT exceeding 10 s is generally considered normal, while a TBUT below 5 s has been associated with severe DED^3,4^. TBUT measurement starts immediately upon the eye’s opening and concludes when the tear film breaks^5^. The average of three tests per eye is the TBUT. The calculated TBUT indicates the quality of the tear film as well as the degree of dryness of the eye. While the TBUT test plays a crucial role in assessing dry eye, its clinical utility is challenged by its low reproducibility. This means that the test results can be inconsistent, making it sometimes difficult to accurately determine when the tear film begins to break up^6^.

### Automated TBUT measurement

AI has substantially contributed to ophthalmology in recent years, revolutionizing diagnostic^7–10^, prediction^11,12^, and therapeutic approaches. Specifically, AI-driven DED investigations have experienced significant advancements, characterized by notable progress and a growing number of studies^13^. One of the pioneering studies in automating TBUT measurement was conducted by Yedidya et al.^14^. They developed an algorithm to assess tear film stability by analyzing videos recorded during slit-lamp examinations. Their method utilizes the Levenberg–Marquardt algorithm to detect dry areas. The initial testing involved videos from 8 patients and achieved an accuracy of 91% compared to assessments by an optometrist. Subsequently, the algorithm was expanded to include measurements of TBUT, analysis of tear film thinning, and detection of dryness caused by the meniscus^15^. Several authors have introduced a method for automating TBUT measurement^16–20^. This technique involves the analysis of color information extracted from tear film videos. The frame corresponding to the TBUT is identified by detecting the highest percentage of dark pixels.

While these studies paved the way, they were based on fundamental methods without incorporating AI. Su et al.^21^ conducted the first groundbreaking study using deep learning for TBUT measurement. They used a digital slit lamp to record TBUT measurements for 80 patients. Fifty participants were used to train the Convolutional Neural Network (CNN) model to recognize areas of tear film break-up, while the remaining 30 were used to validate the proposed TBUT measurement method. A correlation r = 0.9 between predicted and observed TBUT was obtained. Vyas et al.^22^ conducted research using 30 TBUT videos collected from a private eye hospital captured through a TOPCON DV3 camera integrated with a slit lamp. They employed a CNN to detect the presence/absence of DED from TBUT videos, achieving an accuracy of 83%. The algorithm also categorized the severity level of DED as normal, moderate, or severe based on TBUT measurements.

Shimizu et al.^10^ conducted a study to estimate TBUT utilizing a smart eye camera to record ocular blue light videos. The research involved the collection of a dataset comprising 79 patients and utilizing the Swin Transformer as an AI model. The diagnostic accuracy was 0.789, with an AUC of 0.877 for breakup estimation from individual frames. A correlation coefficient (r) of 0.791 was also observed between the TBUT values obtained from the proposed AI solution and those derived from Electronic Medical Records (EMR). Abdelmotaal et al.^23^ study aimed to assess the performance of CNNs in automatically diagnosing DED by analyzing frames from videos obtained through videokeratoscopy. The study included a retrospective cohort of 244 ocular surface videos, and the obtained AUC for discriminating normal eyes from eyes with DED was 0.98.

Despite the notable progress in using AI for TBUT estimation in the studies cited above, some limitations exist. Some studies focused solely on severe dry eye and considered TBUT exceeding 5 s as a normal^21^, had smaller datasets than ours^22^, or used less accessible acquisition systems^10,22,23^.

This study aims to address these challenges by developing an AI approach capable to improve the precision and dependability of TBUT measurement from slit lamp videos with a limited number of datasets. Although a three-dimensional (3D) model stands as a straightforward solution to this task of video analysis, its efficacy is compromised when confronted with a limited dataset^24^. Consequently, there is a need for a more precise solution capable of operating effectively with a limited dataset, such as Siamese networks.

### Siamese network

The Siamese network architecture is specifically designed to compare and detect changes between pairs of input data, making it suitable for tasks such as face recognition^25^, scene change detection^26^, and remote sensing change detection^27^. The Siamese network contains two identical subnetworks with the same weights and architecture; each subnetwork independently processes one of the input samples. These subnetworks commonly consist of convolutional layers followed by fully connected layers, although the specific architecture may vary depending on the task. The Siamese network is adopted not only for change detection tasks but also for multi-task learning. In a study by Fan et al.^28^, a methodology for automated glaucoma diagnosis was introduced, leveraging a multi-task Siamese network. This network facilitates the concurrent acquisition of fundus image similarity measurement and glaucoma diagnosis. Additionally, Zhiwei et al.^29^ proposed a method utilizing the Siamese network to classify retinal artery/vein types in vessel segments based solely on visual features. The approach further involves estimating the similarity between connected segments by comparing both their visual and geometric features, enabling the disentanglement of the vasculature into individual vessel trees.

## Methods

### Dataset

This section outlines the systematic approach adopted for dataset acquisition, preparation, and preprocessing, adhering to standard clinical protocols and ethical guidelines. The study protocols were approved by the Brest, France hospital (CHRU), Ethics Committee.

#### Acquisition protocol

The acquisition protocol is designed in alignment with established clinical practice for Tear Break-Up Time (TBUT) and surface staining assessments, crucial for corneal and conjunctival evaluation^30^. The protocol encompasses:

(i) *Illumination and Fluorescein Instillation* To ensure consistent conditions during the clinical examination, all participants were examined under mesopic conditions with room lights turned off. Additionally, the light intensity of the slit lamp was meticulously controlled throughout the study. Utilizing a cobalt blue light with a yellow filter to create a colored disc. A 2% fluorescein dye is instilled into both eyes, ensuring optimal focus and brightness settings. Patients are then instructed to blink three times to distribute the dye evenly across the tear film.
(ii) *TBUT Recording* Concentrating on the right corneal apex, recordings proceed until the start of tear film break-up. This process is replicated thrice for each eye, followed by an analogous procedure for the left eye.
(iii) *Staining Evaluation* Following TBUT measurements, corneal and conjunctival staining are evaluated by an ophthalmologist.

Recordings are captured using the ION Imaging system, engineered by MARCO, and globally distributed by QUANTEL MEDICAL. This study was performed in accordance with the guidelines of the Declaration of Helsinki. Written informed consent was obtained from all participants for inclusion in the study.

#### Dataset preparation and preprocessing

The dataset consists of 67 videos, with 47 allocated for model development including training and validation, and 20 for testing. Each video was recorded at a frame rate of 60 frames per second and a resolution of 1080 $\times$ 1080 pixels, originating from a unique patient and encompasses three TBUT subtests per eye.

**Figure 1:**
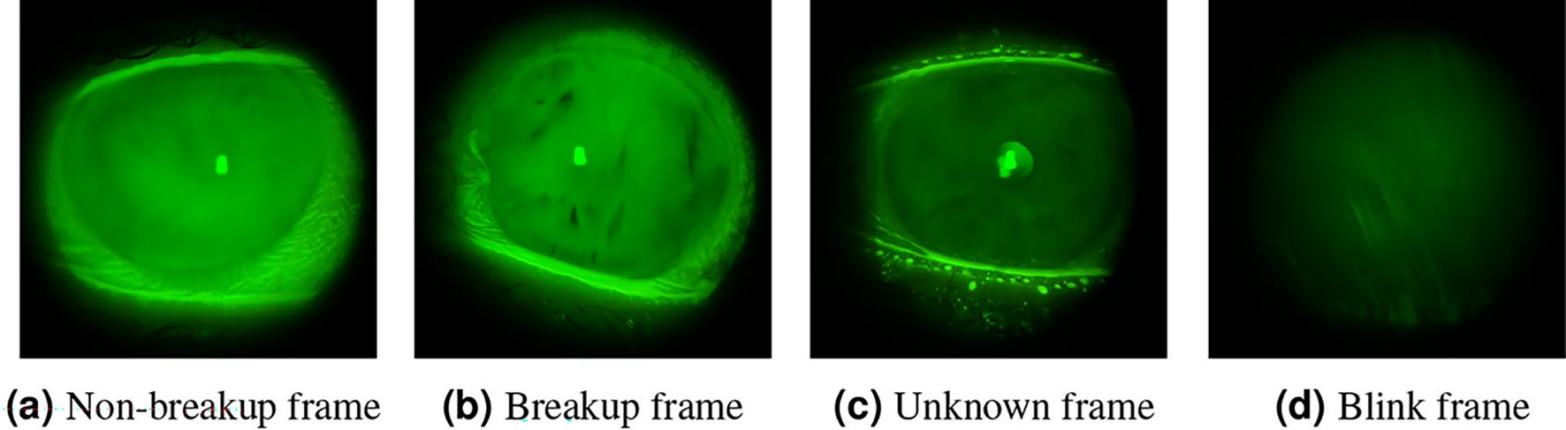
Sample of TBUT video frames from the four different classes.

(i) *Development dataset*
  We conducted frame-by-frame annotations for the 47 videos, which were performed independently by two domain experts, one of whom was an ophthalmologist. We only used annotations where both experts agreed, ensuring consistency in ground truth labeling. These experts were permitted to review the recorded video footage multiple times. Rewinding the recordings enabled meticulous assessment of tear film distribution variations over time, minimizing the potential for oversight and enhancing the reliability of TBUT measurements. A consensus-based annotation strategy was implemented to ensure the objectivity of the dataset, classifying frames into four categories as shown in Fig. 1:
  - *Non-Breakup* Depicting an open eye with a stable tear film.
  - *Breakup* Depicting an open eye, highlighted by the presence of black spots, indicative of a tear film disruption. This condition is identified by the initial emergence of these spots and confirmed through their persistent presence in subsequent images, which establishes the continuity of the tear film breakup.
  - *Unknown* Frames challenging to categorize due to ambiguous indications of tear film breakup initiation.
  - *Blinking* Each sequence of frames captures the entire process of rapid changes in eyelid position and closure, including the period of closed eyelids, and concludes just before the eye returns to a fully open state with stable eyelids.
  For model training, our approach necessitates using frame pairs as input data. Accordingly, we meticulously extracted 3000 frame pairs for each patient. To ensure relevance to the same TBUT measurement and eye, pairs were curated from consecutive frames belonging to the same subtest. However, we incorporated a randomized time interval between selected frames to introduce variability. This methodology allows for the creation of two distinct groups of frame pairs:
  *Similar Pairs* Comprising 50% of the dataset, these pairs consist of frames classified within the same category (either non-breakup/non-breakup or breakup/breakup), ensuring the pairs are homogenous in terms of the tear film’s state.
  *Dissimilar Pairs* Making up the remaining 50%, these pairs are composed of frames from differing categories (breakup and non-breakup), fostering heterogeneity in the training data to challenge and refine the model’s classification capabilities.
  This balanced distribution of similar and dissimilar pairs is critical for training our model to accurately discern between the nuanced states of tear film integrity. For development, frames classified as “unknown” or “blink” were systematically excluded from this process to maintain the focus on clear indicators of tear film status. For the validation and test datasets, all frames were utilized, irrespective of pair requirements, to ensure comprehensive evaluation capabilities.
(ii) *Test dataset*
  To further enhance the experimental rigor and bridge the gap between automated analysis and clinical practice, an experienced ophthalmologist was enlisted to provide TBUT measurements for the 20 videos in the test dataset. This procedure was meticulously conducted within the same clinical environment as standard patient evaluations in the real-world scenario, where the ophthalmologist calculated TBUT measurements in real-time, as they would during a typical clinical assessment.
  In addition to that, the test dataset was also annotated frame-by-frame by a different expert, establishing a solid ground truth for the model’s evaluation.
  This unique approach facilitated a direct comparison between the AI model’s performance and the ophthalmologist’s real-time assessments, against the frame-by-frame analyzed ground truth. The aim was to rigorously assess the AI model’s accuracy, not only against the meticulously annotated ground truth but also in comparison to traditional clinical TBUT evaluations conducted by ophthalmologists.
(iii) *Preprocessing and data augmentation*
  In the preprocessing stage, frames are resized to match the default input dimensions required by each pre-trained model. Additionally, we employed Randaugment, a robust method that randomly applies a subset of predefined augmentations to the original training images, thereby enhancing the model’s robustness to input variations^31^.

The devised multistep approach involves initially employing a Siamese network for dual-task learning. These tasks encompass change detection between pairs of frames and frame classification. Subsequently, upon acquiring frame predictions to discern between breakup or non-breakup tear films, a smoothing technique is applied to refine the temporal predictions, ultimately yielding TBUT.

### Dual task siamese network

The fundamental concept behind a Siamese network is to learn a representation of the inputs within a shared feature space. This space position inputs with similar characteristics closer together and those dissimilar farther apart. In our case, this is achieved through minimizing contrastive loss^32^ during training. Due to our dataset’s limited number of patients, we faced challenges assessing and preventing overfitting while training a convolutional neural network (CNN) classifier. To address this limitation, we designed a Dual-Task Siamese Network (DTSN) architecture. Our DTSN quantifies the similarity between paired frames and classifies each frame as indicative of a breakup event. Moreover, our adoption of multi-task learning effectively mitigates overfitting by encouraging the model to generalize across related tasks^33^. This approach aligns with prior research in Siamese neural networks, as extensively explored in notable studies^34^. In our study, the proposed approach undertook two tasks.

**Figure 2:**
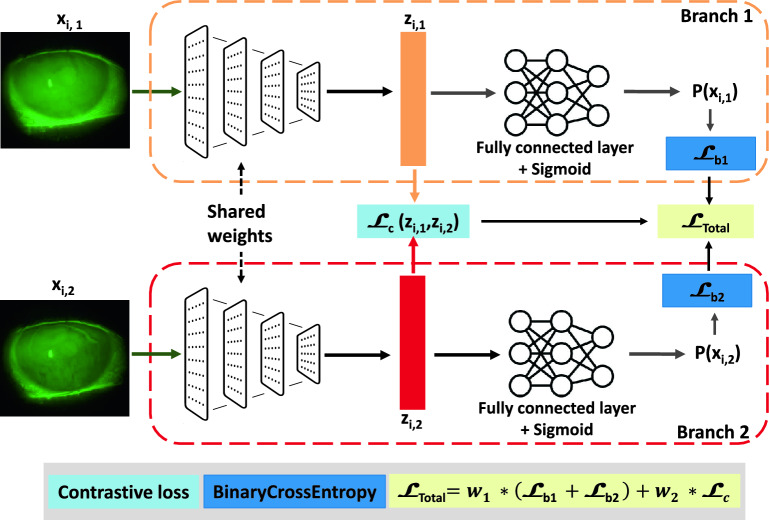
The proposed Dual Task Siamese Network (DTSN) architecture for frames classification: It consists of two identical subnetworks; each pair of frames, $(x_{i,1}, x_{i,2})$, is passed through these subnetworks, resulting in two feature vectors, $z_{i,1}$ and $z_{i,2}$, respectively. The contrastive loss $\mathscr {L}_{c}$ is then computed based on the Euclidean distance between $z_{i,1}$ and $z_{i,2}$, indicating the similarity between $x_{i,1}$ and $x_{i,2}$. ${\mathscr {L}}_{b}$ is a binary cross entropy (where $b \in \{1,2\}$ is the branch index). In the validation phase, only one branch of the networks was used as a non-breakup/breakup classifier.

The primary task involved training the network to classify frames as a breakup or non-breakup. The secondary task is change detection, which requires comparing pairs of frames and involves the network in learning visual features. The model architecture shown in Fig. 2 consists of two identical subnetworks; each pair of frames, $\left( x_{i,1}, x_{i,2}\right)$, $1 \le n \le N$, is passed through these subnetworks, resulting in two feature vectors, $z_{i,1}$ and $z_{i,2}$, respectively. The contrastive loss ${\mathscr {L}}_{c}$ is then computed based on the Euclidean distance between $z_{i,1}$ and $z_{i,2}$, indicating the similarity between $x_{i,1}$ and $x_{i,2}$. To extract relevant features from the video frames, we explored multiple pre-trained networks on ImageNet^35^ as a backbone. The DTSN is trained by minimizing the weighted total loss ${\mathscr {L}}_{Total}$ as follows:

1 $$\begin{aligned} {{\mathscr {L}}_{Total}} = w_1 \cdot ({{\mathscr {L}}_{b1}} + {{\mathscr {L}}_{b2}}) + w_2 \cdot {{\mathscr {L}}_{c}} \end{aligned}$$

$w_1$ and $w_2$ are the applicable weights to the losses: $w_1$ for the classification task, and $w_2$ for the change detection task. After hyperparameter optimization, we fixed $w_1$ to 0.9 and $w_2$ to 0.1. ${\mathscr {L}}_{b1}$ and ${\mathscr {L}}_{b2}$ are both a BCE loss (${\mathscr {L}}_{b}$, $b\in \{1, 2\}$).

2 $$\begin{aligned} {{\mathscr {L}}_{b}} = -\frac{1}{N} \sum _{i=1}^{N} \left[ y_{i,b} \cdot \log ({p}_{i,b}) + (1 - y_{i,b}) \cdot \log (1 - p_{i,b})\right] \end{aligned}$$

where *N* is the number of samples in the dataset. $y_{i,b}\in \{0, 1\}$ is the true label or ground truth for *b*-th element of the *i*-th sample. It is either 0 or 1, representing the two classes “non-breakup” and “breakup” respectively. $p_{i,b}$ are the predicted probabilities.

3 $$\begin{aligned} {{\mathscr {L}}_{c}} = \frac{1}{N} \sum _{i=1}^{N}{(1 - Y_i) \cdot ||z_{i,1} - z_{i,2}||^2 + (Y_i) \cdot \max ({margin} - ||z_{i,1} - z_{i,2}||, 0)^2} \end{aligned}$$

where $Y_i\in \{0, 1\}$ is the pair’s label. A label of 0 indicates similarity between the pairs, while a 1 indicates dissimilarity ($Y_i = 1 \Leftrightarrow y_{i,1} \ne y_{i,2}$). The margin defines a radius around the embedding space of a sample so that dissimilar pairs only contribute to the contrastive loss function if the Euclidean distance $||z_{i,1} - z_{i,2}||$ is within the margin. After hyperparameter optimization, where we chose the hyperparameter values that maximize cross-validation scores, we fixed the margin value at 0.01 as shown in Table 1.

**Table 1:** The hyperparameter values used for DTSN.

Hyperparameter	Optimizer	Learning rate	Schedular	Batch size	Margin	Epochs
Value	Adam	0.0001	StepLR	32	0.01	40

To ensure rigorous evaluation, we applied a fivefold cross-validation approach. Our development dataset was divided into fivefolds, maintaining a consistent ratio between positive and negative samples in each fold. Each fold’s performance was evaluated separately on the test set, and the final AUC was obtained by averaging the AUC of the fivefolds. In the validation phase, only one branch of the network was used as a non-breakup/breakup classifier.

### From frame classification to breakup time estimation

**Figure 3:**
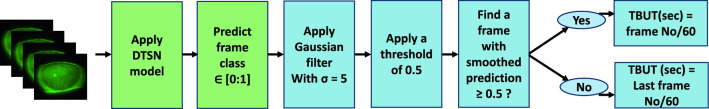
Pipeline for TBUT estimation after frames classification. Green boxes denote actions applied at the frame level, while blue boxes signify actions applied to sequences.

In the inferential processing pipeline illustrated in Fig. 3, sequential analysis is conducted on each frame within a subtest. Our developed model is then used to classify all the frames. A Gaussian filter with a standard deviation $\sigma$ of 5 (determined through hyperparameter optimization) is applied to improve precision. This step effectively mitigates noise and reduces inconsistencies leading to better filtering of outliers in TBUT estimation and allowing the incorporation of temporal information in the decision-making process.

A decision threshold of 0.5 is established. TBUT initiation is identified when the smoothed prediction of a frame surpasses or equals the threshold. In such cases, TBUT in second is determined by the frame number divided by the frame rate (60). If no frame exhibits a smoothed prediction greater than or equal to 0.5, the last frame number divided by 60 is taken as the TBUT. This systematic approach is applied to all subtests within the videos. The meticulous methodology and framework employed herein provide a robust basis for the accurate measurement of TBUT.

## Results

### Performance evaluation using different pre-trained CNNs as a DTSN backbone

Our investigation employed a Dual-Task Siamese Network (DTSN) to classify TBUT frames into non-breakup and breakup categories. Evaluation of various pre-trained models revealed that InceptionV3, EfficientNetB3NS, and RexNet150 delivered the most compelling results, as shown in Table 2. Specifically, RexNet150 was distinguished for its superior efficiency, evidenced by a reduced number of parameters and Floating Point Operations (FLOPs), which translates to faster inference speed and a smaller memory footprint. This selection was validated by achieving an AUC value of 0.870 in the test set, leveraging a combination of predictions from five models through cross-validation. Table 2 showcases the classification performance of different backbones within our DTSN framework on the validation dataset. It highlights the balance RexNet150 strikes between model complexity and performance, making it an optimal choice for our objectives.

**Table 2:** Classification performance comparison of different backbones for DTSN on the validation dataset.

Method	Backbone	Variant	Resolution	Parameters	FLOPs	Validation AUC
DTSN	ResnetV2	Resnet50V2	224	25 Million	4 Billion	0.790 ± 0.03
	Vision transformer	vit_base_patch16_224	224	87 Million	67 Billion	0.831 ± 0.04
	Densnet	Densnet121	224	8 Million	4 Billion	0.894 ± 0.04
	Inception	InceptionV3	224	24 Million	7 Billion	**0.906** ± **0.06**
	EfficientNet	EfficientNetB3NS	300	12 Million	2 Billion	**0.907** ± **0.05**
	RexNet	RexNet150	224	**10 Million**	**1 Billion**	**0.901** ± **0.05**

Significant values are in bold.

The results were contrasted with the performance of the same DTSN architecture but without pretraining on ImageNet, resulting in an AUC of 0.74 on the validation set. Additionally, the results were compared with those derived from a baseline model, specifically a CNN employing BCE loss, resulting in an AUC value of 0.771. To assess the efficacy of the proposed DTSN approach, a comparative analysis was conducted against one of the latest state-of-the-art methods^10^, as delineated in Table 3.

**Table 3:** Performance evaluation for the baseline and the proposed DTSN model pre-trained on ImageNet and comparison with a state-of-art approach.

Method	Backbone	Loss	AUC
Baseline (CNN model)	RexNet150	BCE loss (Eq. 2)	0.771
Shimizu et al.^10^	Swin transformer	BCE loss (Eq. 2)	0.754
Ours (DTSN)	RexNet150	Proposed loss (Eq. 1)	**0.870**

Significant values are in bold.

### TBUT estimation

**Figure 4:**
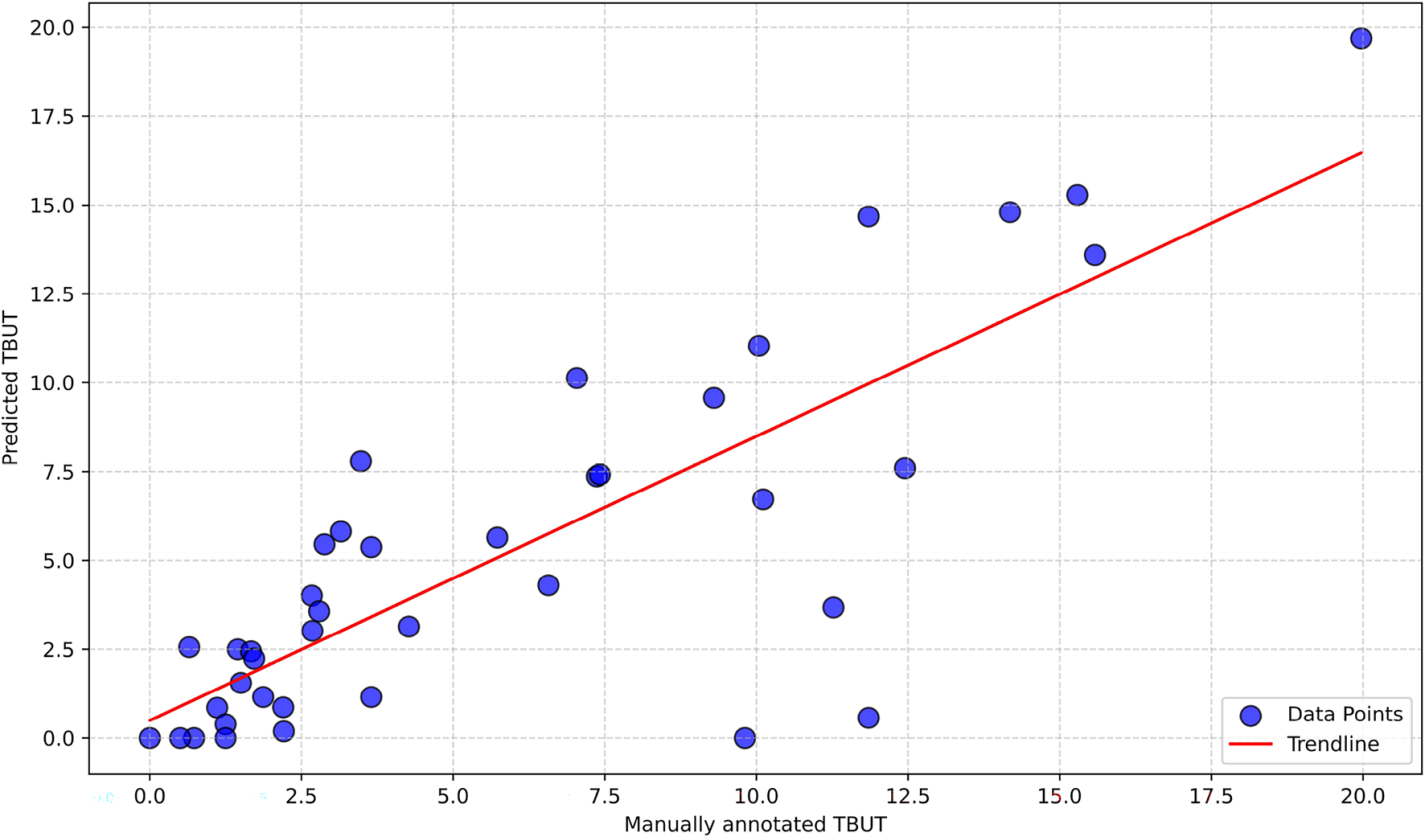
Correlation between manually annotated TBUT and estimated TBUT with AI.

As presented in “2.1” section, the test set encompasses 20 TBUT videos, meticulously annotated frame-by-frame, serving as our designated ground truth and also with an ophthalmologist in real-time. Our developed algorithm was applied to predict TBUT values for each video in the dataset. As illustrated in Fig. 4, a robust Pearson correlation coefficient r = 0.81 was computed between the TBUT assessment conducted by the proposed approach and the ground truth. Certain outliers, such as the two data points at the bottom, exhibit significant differences. In these cases, the predicted TBUT values are 0 s and 0.57 s , while the corresponding ground truth values are 9.81 s and 11.85 s, respectively. In these particular instances, post-blinking and upon opening the eyes, residual black spots persist due to inadequate fluorescence spreading on the ocular surface. The model interprets these black spots as indicative of the initiation of tear film breakup.

**Figure 5:**
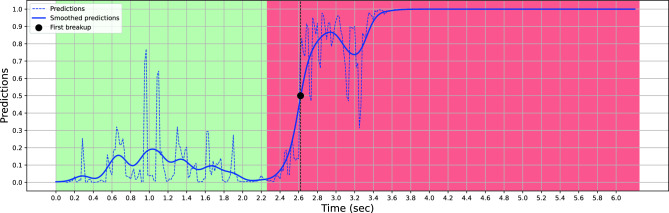
Comparison of Tear Film Dynamics for a given patient: Ground Truth and Predictions.

Figure 5 depicts the dynamics of a subtest for a given patient in the test set. The figure consists of two distinct curves and two different background colors representing various aspects of tear film characteristics. The green and red backgrounds represent the ground truth, where one corresponds to the normal tear film state when the label is 0, and the other represents the breakup of the tear film when the label is 1. These ground truths serve as references for evaluating the accuracy of subsequent predictions. The blue dashed line in the figure represents the classification predictions obtained through our developed model. This curve reflects the model’s ability to accurately classify the tear film state based on the input data. It provides a quantitative estimation of tear film dynamics and allows for comparison with the ground truth data. Predictions are often of good quality at the extremes and noisier at the beginning of the break-up sequence. The blue curve illustrates the smoothed predictions. This curve represents an improved version of the classification predictions. The smoothing process aims to reduce noise or fluctuations in the data, providing a clearer representation of the tear film dynamics. By leveraging the smoothed predictions, it becomes possible to estimate the TBUT, a critical metric in assessing tear film stability.

The estimated TBUT is determined by analyzing the smoothed predictions and identifying the time at which the tear film breakup occurs. As depicted in Fig. 5, the initiation of tear film breakup occurs when the smoothed prediction value surpasses or equals the predetermined threshold of 0.5, denoted by a black marker in the graphical representation. This figure and its accompanying curves offer a comprehensive visual representation of tear film dynamics. They demonstrate the relationship between ground truth data, classification predictions, smoothed predictions, and the subsequent estimation of TBUT.

**Figure 6:**
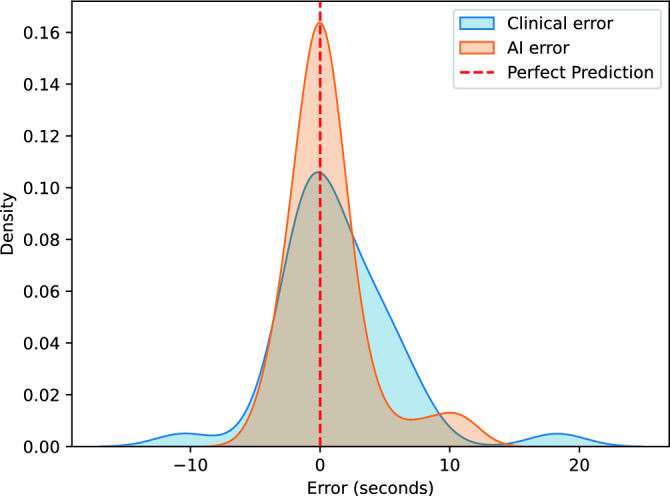
The error density for AI and clinical predictions versus ground truth.

The mean error observed between the annotations provided by two experts on the development dataset, at the level of breakup detection, stands at 0.36 ± 1.31 s. This underscores the significance of interoperator variability in determining the precision of estimated TBUT. Figure 6 shows the error density for AI and clinical predictions versus ground truth within the test dataset. The AI model has a more concentrated error distribution around zero, indicating better accuracy.

### Performance evaluation using different frame rates

To investigate the influence of frame rate on tear film breakup detection accuracy, we conducted additional experiments where we systematically varied the frame rate during both the development and testing phases of our AI model. Interestingly, the performance between 60 frames per second (fps) and 30 fps showed negligible difference. However, as shown in the table below, frame rates below 30 fps lead to a significant decline in model performance.

**Table 4:** Method’s performance evaluation using different frame rates.

Frame rate (fps)	Breakup detection AUC	Mean error (s)	Correlation coefficient r
60	0.870	0.731	0.814
30	0.868	0.736	0.810
15	0.779	2.370	0.577
5	0.747	3.541	0.487

## Discussion and conclusion

DED is a prevalent chronic condition characterized by tear film instability, predominantly caused by either inadequate tear production or excessive tear evaporation. The TBUT test is commonly used for DED diagnosis but is subjective, time-consuming, and labor-intensive. Moreover, conventional computer-aided DED detection techniques rely on expensive imaging instruments that are not universally accessible. Developing a DED detection approach also faces challenges such as poorly illuminated videos, constant eye blinking, blurred imagery, and a lack of public datasets. In this context, our study introduced a Dual-Task Siamese Network architecture specifically designed for simultaneous classification and change detection tasks.

The classification task involved labeling video frames as either non-breakup or breakup, while the change detection task aimed to compare pairs of frames to identify similarities or dissimilarities. By implementing this approach, we achieved promising results, including an AUC of 0.870 for the classification task and accurate estimation of TBUT values for patients in the test set. The empirical evidence, underscored by a Pearson correlation coefficient of 0.81 between AI-derived and ground-truth TBUT estimations, firmly establishes the superior accuracy of our AI model over traditional clinical assessments.

The time of evaluating TBUT using our proposed network can be delineated into distinct stages. Initially, the extraction of the subtest, comprising frames between two blinking sequences where the eye is fully open and the eyelid is stable, was performed manually in our study. While this process can be automated through the classification of blinking and open-eye frames, our primary focus lies on predicting TBUT rather than optimizing this extraction step. Once the subtest is identified, our method requires 1 s per frame to detect the break-up and predict TBUT using Nvidia RTX A5000 GPU. It is essential to note that our approach prioritizes the efficiency of TBUT estimation rather than mitigating time-consuming limitations in the evaluation process. This consideration is pivotal for future improvement to integrate our method into clinical practice, offering a more precise and reliable tool for ophthalmological assessments, and objectively addressing the limitations encountered in conventional measurement techniques.

Our choice of utilizing a Siamese network architecture proved to be effective in accurately classifying non-breakup and breakup classes compared to a single CNN. The network’s ability to learn shared feature representations allowed it to discern subtle differences between the two classes, resulting in a high AUC value. This performance indicates the robustness of the network in the classification of the frame. Additionally, a Siamese network was introduced as an effective countermeasure to combat overfitting while enhancing CNN’s performance. Using pre-trained neural networks as a backbone for feature extraction played a crucial role in achieving our results. Leveraging the knowledge captured by these networks allowed us to extract relevant features from the video frames effectively. Our choice of using BCE loss for the classification task and contrastive loss for the change detection task was also pivotal. The BCE loss facilitated the network’s training to accurately classify frames as non-breakup or breakup, while the contrastive loss function enabled the network to distinguish between similar and different pairs of frames. This combination of loss functions allowed our network to extract discriminative features and learn representations that successfully captured the variations present in the data. Furthermore, our approach demonstrated its capability to estimate TBUT values accurately.

As demonstrated in Table 4, reducing the frame rate below 30 fps leads to a significant reduction in performance. Higher frame rates generate larger datasets by capturing more frames within the same recording duration. This increased volume of data provides richer temporal information, which is crucial for training our AI model more effectively. More comprehensive temporal sampling allows the model to learn from a broader range of subtle dynamics and variations in the tear film, which are essential for accurately estimating TBUT. Consequently, maintaining a minimum frame rate of 30 fps is critical for optimizing model training and performance.

The model’s predictions demonstrated a comparable range of values to the ground truth. However, disparities between the model’s predictions and the manually annotated TBUT were observed in certain cases, resulting in discrepancies regarding the severity level. Therefore, further refinement and evaluation of the model may improve its accuracy and align it more closely with expert assessments in diagnosing tear film stability disorders to use it as a valuable tool in clinical settings, and to provide objective and consistent measurements of TBUT values. A possible advantage of using the frame sequence approach [DTSN + Long Short Term Memory (LSTM)] is that it considers the temporal dynamics depicting changing frame features with time as caused by tear film breakup.

This study demonstrates the robust performance of the proposed AI approach for quantifying TBUT. However, our study had several limitations. First, while the number of frames and pairs was relatively large, the study included a limited number of eyes and corresponding videos. Incorporating an expanded dataset promises to refine and optimize our proposed TBUT quantification approach in future investigations. Another limitation was that our dataset was selected from one institute. Thus, any generalizations from our findings should take this feature into account.

In conclusion, our study comprehensively investigates utilizing a Siamese network for dual-task learning in tear film analysis. By leveraging shared feature representations and employing pre-trained networks and different loss functions, we achieved outstanding results in both the classification task and the estimation of TBUT values.

## Data Availability

The data used to train and test our models are not publicly available due to restrictions. They were obtained through labor-intensive and expensive processes and are proprietary. However, they are available from the corresponding author upon reasonable request and with the permission of CHRU Brest and Théa.
